# Human Serum Transferrin Fibrils: Nanomineralisation in Bacteria and Destruction of Red Blood Cells

**DOI:** 10.1002/cbic.201402458

**Published:** 2014-12-04

**Authors:** Arindam Mukherjee, Mark A Barnett, V Venkatesh, Sandeep Verma, Peter J Sadler

**Affiliations:** [a] Department of Chemistry, University of WarwickGibbet Hill Road, Coventry CV4 7AL (UK); [b] Department of Chemistry, Indian Institute of Technology KanpurKanpur 208016 (UP) (India)

**Keywords:** atomic force microscopy, fibrillation, nanomineralisation, red blood cells, siderophores, transferrin

## Abstract

Fibrils formed by human serum transferrin [(1–3 μm) apo-Tf, partially iron-saturated (Fe_0.6_-Tf) and holo-Tf (Fe_2_-Tf) forms], from dilute bicarbonate solutions, were deposited on formvar surfaces and studied by electron microscopy. We observed that possible bacterial contamination appears to give rise to long, pea-pod-like (PPL) structures for Fe_2_-Tf, attributable to the formation of polyhydroxybutyrate (PHB) storage granules, under the nutrient-limiting conditions used. These PPL structures contained periodic nanomineralisation sites susceptible to uranyl stain. Extended incubation of transferrin solutions (about four days) gave rise to extensive transferrin fibril structures. Optical microscopy and AFM studies showed that red blood cells (RBCs) readily adhere to these fibrils. Moreover, the fibrils appear to penetrate RBC membranes and to induce rapid cell destruction (within about 5 h). It is speculated that in situations in vivo where transferrin fibrils can form, such interactions might have adverse physiological consequences, and further studies could aid the understanding of related pathological events.

## Introduction

Human serum transferrin (Tf) is an 80 kDa bilobal iron-transport glycoprotein, which circulates in blood at a concentration of about 35 μm. In blood, Tf is only ≈30 % saturated with iron (ferric iron, Fe^3+^) and its half-life in the body is about seven to eight days.[1] Fully iron-saturated Fe_2_-Tf binds to transferrin receptors on cells and is taken up by endocytosis. An interesting feature of Fe^3+^ binding is the requirement for a (bi)carbonate synergistic anion, which coordinates to each Fe^3+^ ion in its interdomain binding site. The other four ligands that complete the octahedral coordination are recruited from protein side chains (2×Tyr, His and Asp).

There is much current interest in the flexibility of protein structures and in particular in aggregation, which can have serious physiological consequences: amyloid formation, for example.[2]

Previously we have shown that Tf can form fibres while standing in solution and we have studied their morphology on various surfaces, such as formvar-coated EM grids and mica, by several microscopy techniques.[3] However, precise conditions that support fibre formation, and how they could be related to the nature of Tf samples, were not clear. In particular, structures containing aggregated transferrin as models for the curious pea-pod-like fibres (containing periodic electron-dense deposits, some of which contained iron) were difficult to construct. In subsequent atomic force microscopy (AFM) experiments,[2a] we demonstrated that the Tf structure is indeed flexible, because it can undergo significant flattening when deposited on mica surfaces. Interestingly, aggregation into ordered rounded structures also appeared to be related to pre-dimerisation of Tf in solution.

In this work, we have made detailed investigations concerning the dependence of fibre formation on surfaces by Tf on the extent of iron loading of transferrin, by comparing apo-Tf, partially-saturated Tf and holo-Fe_2_Tf. We have also studied the dependence of fibre formation within a particular batch of transferrin provided by a commercial supplier. This led to the discovery of effects of Tf on bacterial growth and ensuing morphologies, and to interesting effects of the protein on red blood cells. Unambiguous identification of nanometre-sized objects imaged on electron microscope grids by TEM was difficult, but we have made considerable effort to distinguish between possible microbial presence as contamination and protein deposits.

## Results

### Deposition of transferrin on surfaces

We first investigated the morphology of deposits formed by human transferrin dissolved at various concentrations in sodium bicarbonate solution that was allowed to stand for various periods of time after deposition on carbon-coated formvar surfaces of copper TEM grids. Six different batches of transferrin, all commercial and supplied by Sigma–Aldrich, were used (Table 1). They differ as follows: samples **1**, **3**, **4** and **6** are fully iron-saturated (Fe_2_Tf), sample **2** is apo-Tf, and sample **5** is saturated with iron to a similar level as circulating Tf (about Fe_0.6_Tf). Samples **3** and **6** are different batches of the same material (Table 1).

**Table 1:** Description of human serum transferrin samples supplied by Sigma–Aldrich.

Transferrin Cat no./	Sample	Supplier description
lot no.	no.	
holo-transferrin	**1** (Fe_2_Tf)	≥98 %; iron content 1100–1600 μg g^−1^
T4132/038K1107		
apo-transferrin	**2** (Tf)	≥98 % (agarose gel electrophoresis);
T1147/068K1571		iron content ≤0.005 %
holo-transferrin	**3** (Fe_2_Tf)	≈98 % (agarose gel electrophoresis),
T3400/68F_9468		iron-saturated
holo-transferrin (lot	**4** (Fe_2_Tf)	heat-treated at 60 °C (minimum) for
no. T0665/107K1182)		a minimum of 10 h, iron-saturated
human transferrin	**5** (Fe_0.6_Tf)	endotoxin level <1.5 ng g^−1^ of
T6549/24H9310		protein, iron content 30 %
human transferrin	**6** (Fe_2_Tf)	≈98 % (agarose gel electrophoresis),
T3400/14H9320		iron saturated

**Figure 1:**
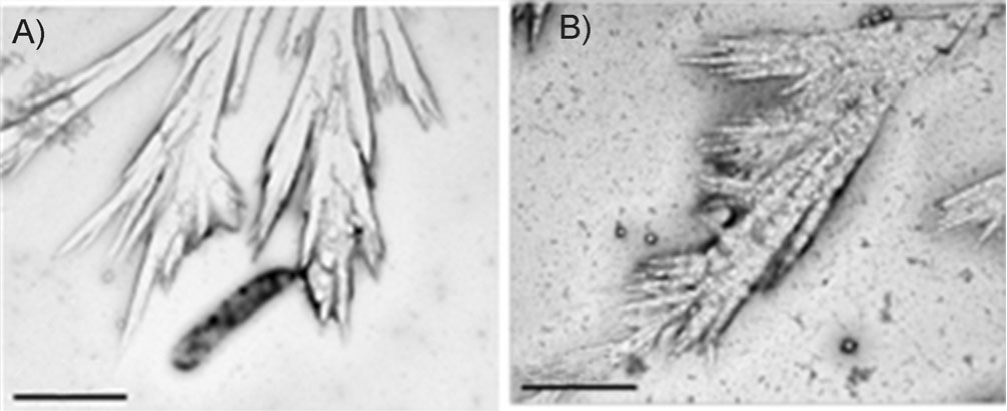
Sample 1 (T4132) after 72 h incubation showing thin fibrillar shapes along with some sodium bicarbonate microcrystals on the fibres. Scale bars: A) 2 μm, B) 1 μm.

TEM pictures of sample **1** (Fe_2_Tf, 3 μm in 3 mm NaHCO_3_), were obtained after incubation at 37 °C for 0–48 h, followed by deposition on formvar. Microscopy images showed only PPL structures containing electron-dense deposits, similar to those we had previously observed (Figure S2 in the Supporting Information).[3] However, a longer incubation of 72 h afforded formation of branched fibre-like growth patterns, as visualised by TEM images (Figure 1).

**Figure 2:**
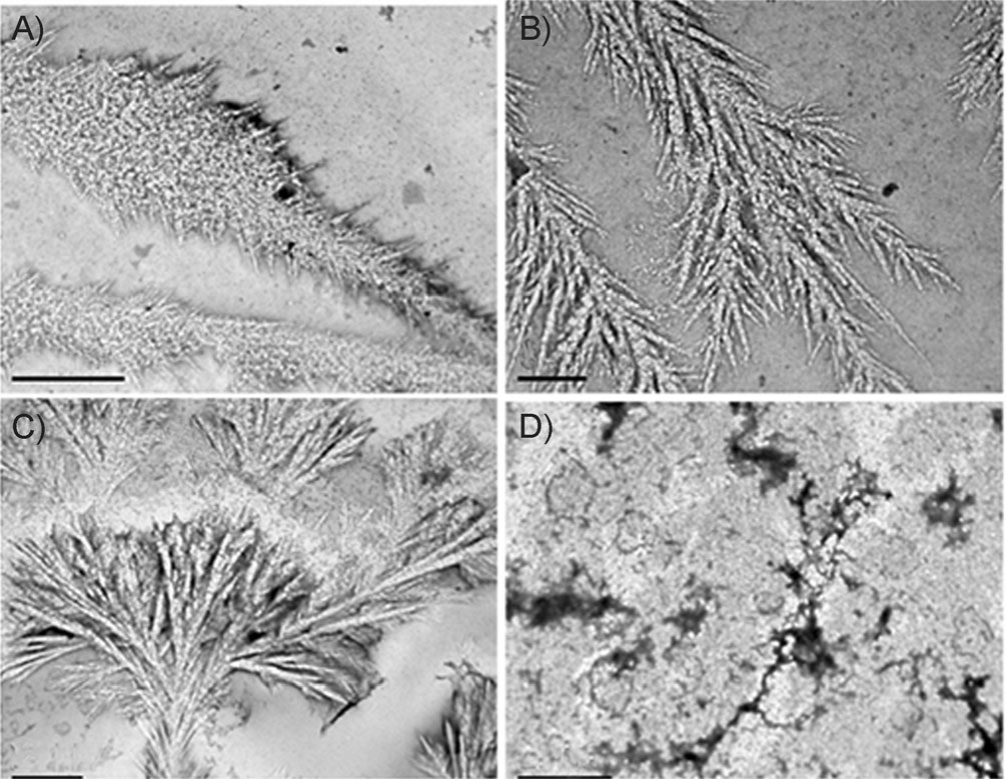
TEM images of 2 (T1147) in 3 mm NaHCO_3_ at 37 °C A), B) after incubation for 24 h, and C), D) after incubation for 72 h, showing the thin fibre-like morphologies. Scale bars: A), C), D) 2 μm, B) 0.5 μm.

A solution of apo-transferrin sample **2** (3 μm in 3 mm NaHCO_3_) was incubated at 37 °C for 24 h. Microscopy analysis showed thin fibre-like growth features with accompanying deposits of salt crystals (Figure 2 A and B). Their appearance remained unchanged after 72 h of incubation, affording a highly branched, dendritic morphology (Figure 2 C), along with some protein precipitate (Figure 2 D). The absence of PPL deposits from this sample suggested that the presence of iron in transferrin is required to promote their formation. This was further confirmed through the studies of two more batches of Fe_2_Tf.

**Figure 3:**
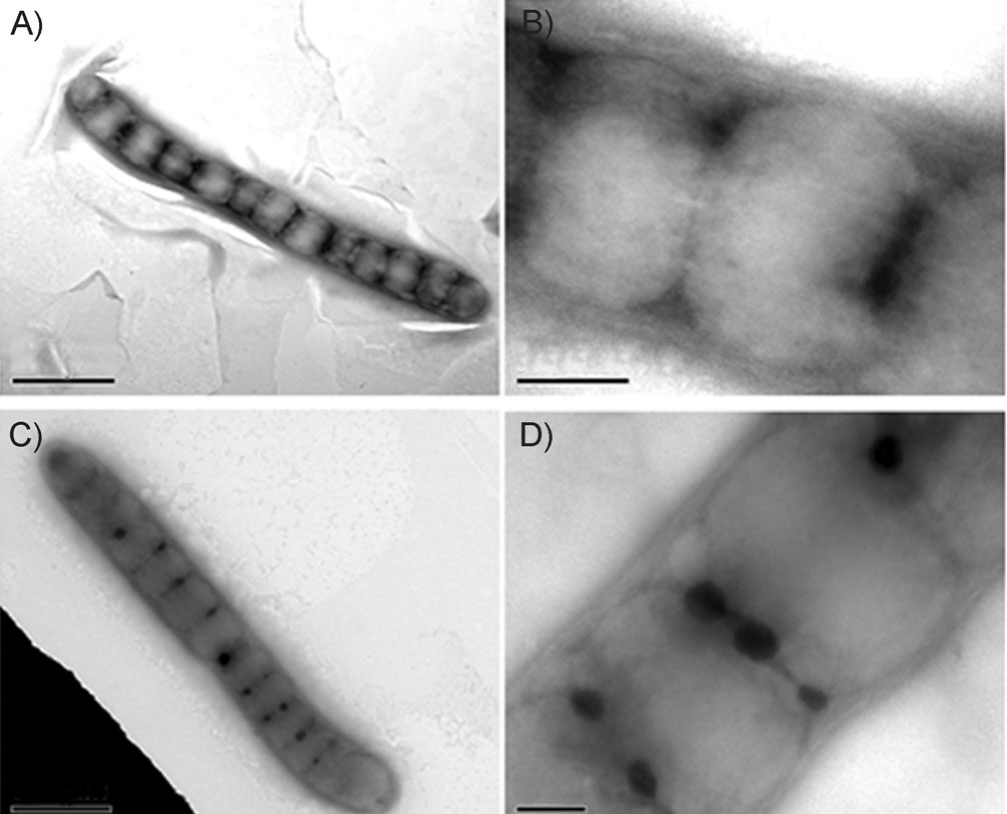
TEM images of 3 (T3400, Sigma) in 1 mm NaHCO_3_ at 37 °C after incubation for 48 h: A), B) incubation at pH 7.4, and C), D) incubation at pH 8.5, showing bacteria-like morphologies of about 500 nm in diameter with periodic spots. Scale bars: A), C) 1 μm, B), D) 0.2 μm.

Sample **3** was diluted to 1 μm (1 mm NH_4_CO_3_, pH 7.4) and incubated at 37 °C. After 48 h, a 6 μL portion of this solution was spotted onto a formvar-coated copper grid, stained with 0.5 % uranyl acetate, washed thoroughly, and dried. TEM images showed prominent PPL structures of widths of about 400–500 nm and lengths of about 10–15 μm or more (Figure 3 A and B). They contained dark periodic bands/spots spaced at intervals of about 300 nm along the length. When the initial sample pH was 8.5, incubation for 48 h gave similar TEM images (Figure 3 C and D), except that the dark bands were more resolved into distinct periodic spots. Energy-dispersive X-ray analysis (EDX) of the dark spots showed the presence of uranium (from the uranyl stain), but no iron (Figure S3).

There appears to be no other precedent for protein fibres with this type of PPL structure in the literature. On the other hand, there are a few reports of bacteria that display related morphologies.[4] Thus, it could be considered that these Tf PPL structures might possibly arise from bacterial contamination of the samples, leading to unusual Tf solution-phase morphologies.

**Figure 4:**
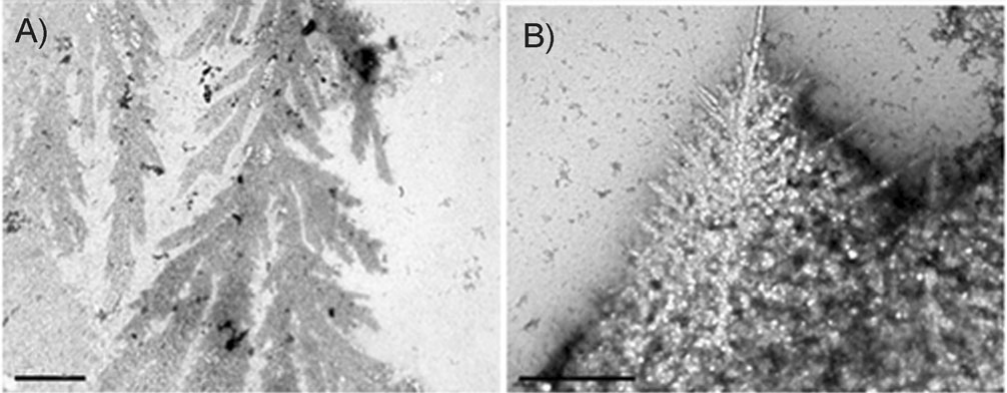
TEM images of sample 3 holo-transferrin (3 μm in 3 mm NaHCO_3_) after incubation for 24–72 h at 37 °C. A) Fern-like fibrillar growth, scale bar: 2 μm, and B) thin fibre shapes with salt crystals, scale bar: 1 μm.

**Figure 5:**
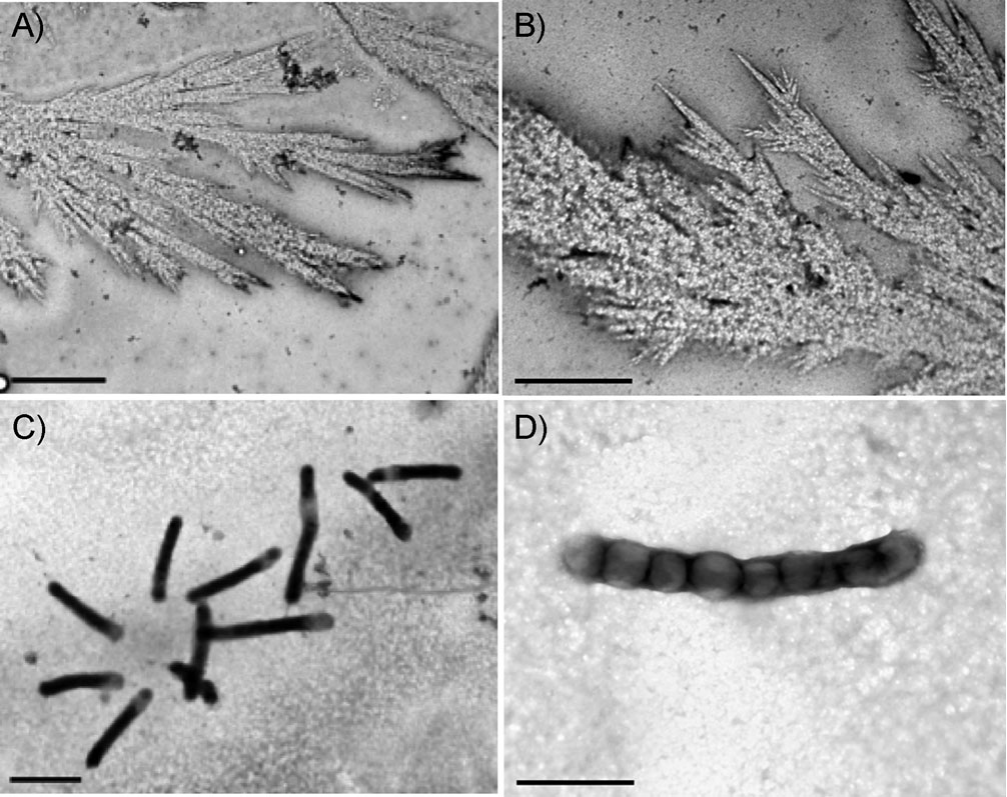
TEM images of sample 6 holo-transferrin (3 μm in 3 mm NaHCO_3_) after incubation for 24–72 h at 37 °C. A), B) Thin fibre-like growths with salt crystals on them, and C), D) tubular shapes with dark bands. Scale bars: A), C) 2 μm, B), D) 1 μm.

In order to probe bacterial contamination, we prepared solutions of sample **3**, followed by incubation for 24 h at 60 °C, to curtail bacterial growth. TEM studies of this sample showed globule-like shapes along with fine networked fibrillar precipitates (Figure S4). The latter structures might be precursors of PPL structures; they also suggest that incubation itself was not solely responsible for such morphologies (or that they are thermophilic bacteria). Incubation of the solution of sample **3** at 37 °C for about 48–72 h did give rise to some thin fibrillar deposits (Figure 4) similar to those seen with sample **1**. Sample **6** also gave PPL deposits on the grids (Figure 5).

### Bacterial growth in transferrin samples

TEM data suggested that commercial transferrin samples might contain bacterial contamination. To confirm this, samples **1**–**6** were streaked onto sterile LB agar plates containing no antibiotic and incubated at 37 °C. We found visible bacterial growth in about 18–48 h. Fe_2_-Tf samples **1**, **3** and **6** gave distinct bacterial colonies in about 18 h (Figure S6 A, C and F), whereas samples **2** (apo-Tf) and **5** (partially iron-saturatedTf, Fe_0.6_-Tf) did not give rise to visible growth within 18 h, although bacterial colonies were observed after about 36 h (Figure S6 B and E). Fe_2_-Tf sample **4** showed bacterial growth only after 48 h at 37 °C (Figure S6 D). Hence, as might be expected, iron saturation of transferrin provides more favourable conditions for bacterial growth except in the case of sample **4**.

Examination of these bacterial colonies by TEM with uranyl acetate staining showed mostly round shapes from the bacteria grown on LB agar plates (Figure S7), distinctly different from the PPL structures obtained above after incubation of iron Tf samples. In comparison, we found that bacteria taken from a non-sterile buffer solution left at room temperature for a week showed long filaments of bacteria with periodic dark bands (Figure S7). These results are in line with the expectation that bacterial growth and morphology are affected by growth, under the conditions of nutrient depletion. The subtle differences between the filamentous form of bacteria, from buffer alone and transferrin samples, show that the observed morphological changes might be due to the influence of transferrin, under nutrient-depleted conditions.

### Interaction of transferrin with red blood cells

Transferrin (Tf) is known to deliver iron for the maturation of red blood cells.[5] The process of cellular iron uptake from transferrin starts with the binding of transferrin to its cognate receptors on the cell surface (through receptor-mediated endocytosis), and the release of iron from Tf then proceeds through endosomal acidification.[6] Early studies carried out with holo-transferrin demonstrated a high affinity for binding to its receptor on the cell membrane at neutral pH.[7]

Red blood cells (RBCs), the most abundant cells in the human body, are also cells with maximum mobility, moving constantly throughout the body and functioning as oxygen carriers.[8] Human erythrocytes are biconcave disks with an average diameter of 6–8 μm and a thickness of about 2 μm. Time-dependent morphological changes of RBCs under physiological buffer conditions have been well reported in the literature.[9] The negative charge on RBCs is known to resist their aggregation and sedimentation, whereas interaction with fibrinogens assists sedimentation. We modelled a situation in which fibres of transferrin might form on a surface in the circulatory system and come into contact with RBCs.

**Figure 6:**
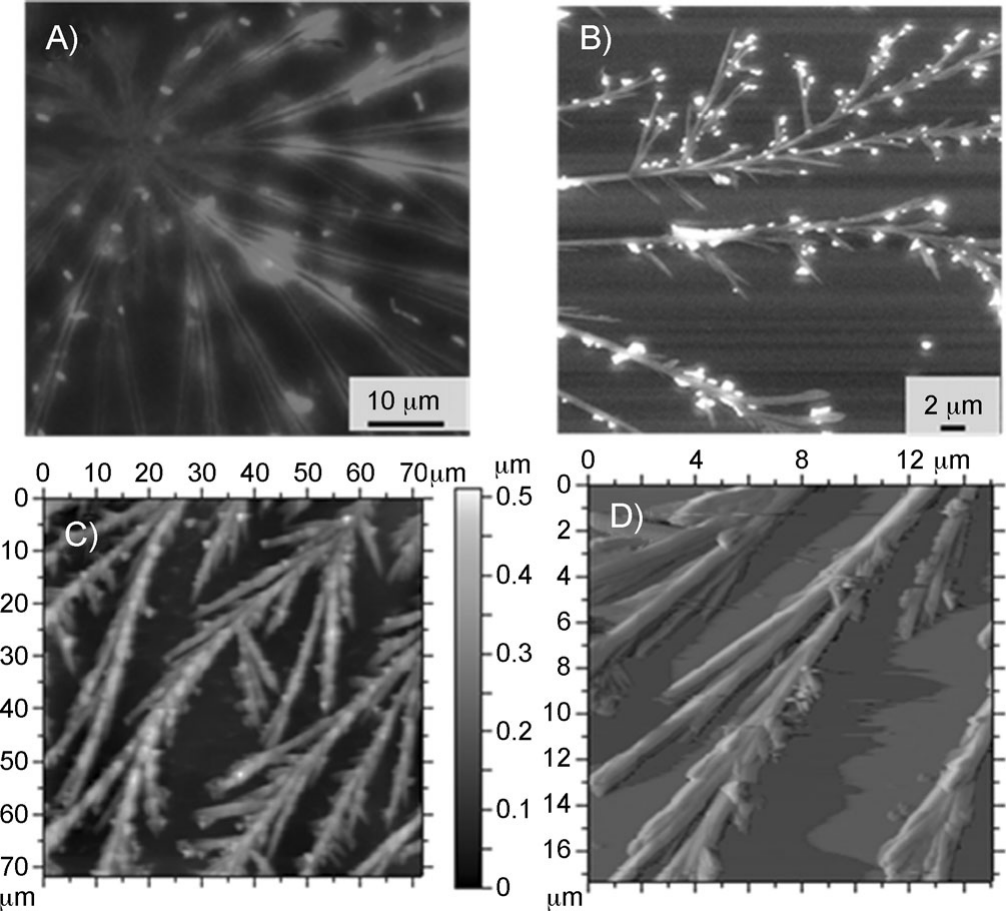
A) Fluorescence optical micrograph of Rhodamine-B-bound holo-transferrin fibres in sodium bicarbonate buffer. B) SEM image of sample 3 holo-transferrin in sodium bicarbonate buffer (aggregates of sodium bicarbonate on transferrin fibres are also visible). C), D) Contact mode AFM images of holo-transferrin in sodium bicarbonate buffer and its magnified view, respectively.

Fresh transferrin solutions of Fe_2_-Tf (sample **3**, 12.5 μm) were prepared in 24 mm sodium bicarbonate buffer (mimicking blood concentration), and a 20 μL aliquot of the sample was spread onto a glass coverslip and allowed to dry at room temperature for visualisation under an optical microscope. After 4 days incubation, a dense fibrillar network was observed. At this concentration, the aggregates formed were not isolated fibre bundles, as was the case for a 1 μm solution of transferrin on the mica surface we studied previously.[3] On staining with rhodamine B, bright red branched fibrils were seen (Figure 6 A). We further confirmed the formation of fibrils by scanning electron microscopy (SEM) and AFM (Figure 6 B and C). In the SEM images, small crystallites of sodium bicarbonate deposited on the surface of the protein fibrils were also clearly observed (Figure 6 B).

**Figure 7:**
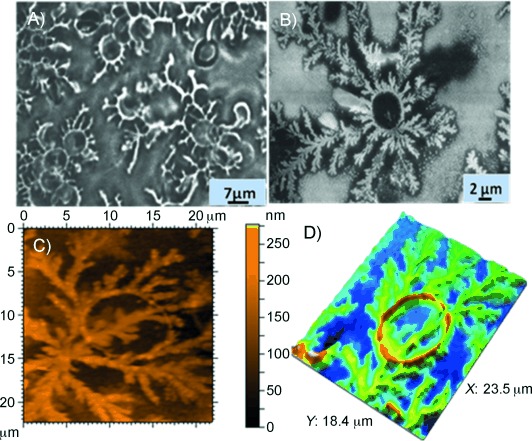
Images of fresh RBCs co-incubated with sample 3 holo-transferrin. A) Optical microscopy, B) SEM, C) contact mode topographic AFM, and D) corresponding 3D micrograph, showing transferrin (fresh solution) that penetrates inside the erythrocyte cell membrane. All the images were taken under dry conditions.

**Figure 8:**
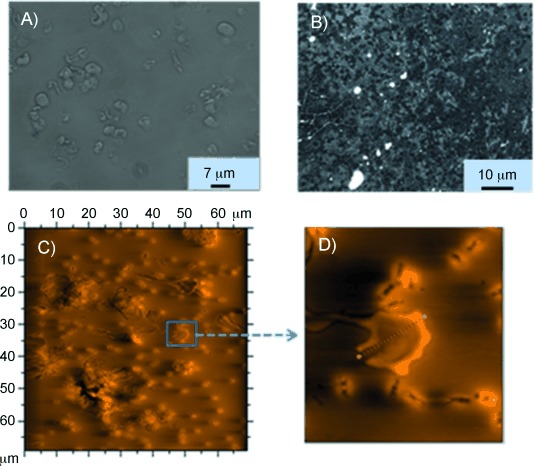
A) Optical microscope image of a 3-h-old RBC-holo-transferrin solution. B) Optical microscope image of 5-h-old RBC-holo-transferrin solution. C) Contact mode AFM image of 1-day-old RBC-holo-transferrin solution showing the leakage of cellular material from RBC. D) Magnified marked area from C).

RBC binding to transferrin fibres was studied by incubating fresh human RBCs with a 4-day-old transferrin solution, followed by visualisation under an optical microscope. Attachment of transferrin fibres to the surfaces of human erythrocytes was observed by optical microscopy (Figure 7 A), followed by AFM (Figure 7 C, D). AFM images of fresh RBCs show biconcave disc shapes (Figure S8). Changes in shape and size of RBCs on co-incubation with transferrin were monitored periodically by optical microscopy and atomic force microscopy. After 3 h, significant changes in the shapes and sizes of the RBCs were observed (Figure 8 A). After 5 h, it was even more difficult to discern the shapes of RBCs (Figure 8 B). An AFM micrograph of a 1-day-old sample revealed leakage of cellular material from RBCs (Figure 8 C).

These observations show that transferrin fibrils initially attach to the RBCs and then penetrate cells, resulting in cell lysis (Figure 8 D). Repetition of these experiments at different concentrations of transferrin (10, 12.5 μm) and buffer (10 μm, 24 mm) resulted in similar observations (Figure S9).

## Discussion

Our results confirmed that transferrin can form fibrils from dilute bicarbonate solutions on carbon-coated formvar surfaces (Figures 1 and 2). At physiologically relevant temperature and pH, serum transferrin can also form networked fibrillar structures. The results also emphasise that PPL structures might not necessarily consist of transferrin protein, but might be bacteria with uranyl deposits (Figure 3). However, we did not detect any iron deposits as we had found earlier in such PPL structures. The morphology of the structures found earlier[3] was very similar but not identical to those found in the present work. Changes in morphology can arise from changes in bacterial texture as a result of changes in factors such as salt concentrations, protein content, nature of the surface, and environment in the locations in which the data were collected (India versus the UK).

There is no evidence in the literature for aggregated protein structures that look like PPL structures, but there are reports of bacteria that do have related morphologies. For example, it has been found that bacteria grown on renewable carbon feedstock or nitrogen-depleted media shows visible deposits of polyhydroxyalkanoates (PHAs) that appear similar to peas in a pod,[10] and their morphology has some similarity to the filamentous structures observed in our experiments. In addition, TEM micrographs of bacteria are also sometimes found to have periodic spots.[11] It is also known that bacteria can exhibit filamentous growth and show asymmetric septa while growing in various media.[12] We found asymmetric septa in some of our PPL structures (Figure 3).

Studies of cultures of bacteria under nutrient-limiting conditions have shown that the permeability barriers of their membranes might be compromised and that the bacteria might enter a non-culturable state.[13] Polyphosphate-deficient cells can become filamentous during the stationery phase of growth and may develop a similar morphology as we observe.[14] In our samples the protein was incubated with bicarbonate at physiological pH without other nutrients. Bacteria in the sample might have their membrane permeability compromised so as to take up transferrin as a nutrient, leading to the filamentous structures with periodic spots or dark bands as we have observed. Intake of transferrin by bacteria is possible because many have transferrin receptors.[15]

Transferrin solutions, when plated onto LB agar dishes in presence of nutrients, gave rise to a bacterial growth pattern with a morphology different from that observed in the TEM images involving only transferrin and a nutrient-depleted medium containing bicarbonate. The TEM images of the bacteria from the transferrin-containing LB agar plates showed bacterial shapes that were mostly discs or round tablet type (Figure S7). In comparison, bacteria from the non-sterile buffer solution without transferrin, but left at 25 °C for a week, showed long filaments of bacteria with periodic dark bands (Figure S7), somewhat similar to our results with holo-transferrin solutions. The results support the conjecture that, under nutrient-depleted conditions, the bacterial growth and morphology (which is found in our case as well) and the observed morphological changes might be due to the presence of transferrin.

When solutions of sample **3** were incubated for 24 h at 60 °C—conditions expected to be unfavourable for bacterial growth—the long shapes were no longer present; rather globule-like, almost spherical shapes and some fine networked fibrillar precipitates formed (Figure S4). These globule-like shapes might be precursors of the PPL structures.

Intriguingly there appear to be nanomineralisation sites for metal ions between the pods. Previously we detected some iron nanominerals that we presumed had formed from iron that had dissociated from holo-transferrin. Perhaps this indicates a route for iron uptake by the starved bacteria. Here, we detected only uranium nanominerals from the uranyl acetate stain used for microscopy staining, which might bind in a similar fashion to iron. The generation of polyhydroxybutyrate (PHB) by bacteria under stress is well known[16] but there appear to be few reported TEM studies of bacteria under such growth conditions. However, it is interesting to note that the uranyl stain shows a selectively higher uptake in certain zones in the bacteria signifying sites of hydroxybutyrate accumulation and polymerisation.

Our studies on the interaction of transferrin fibrils with RBCs therefore focussed on interactions with the long thin fibrous deposits of transferrin. Association of Tf molecules presents an extended binding surface for cells and a potentially iron-rich region. This might lead not only to destabilisation of RBC membranes, but also to opening of the iron-binding clefts of transferrin (due to carbonate release). The liberated iron might initiate free radical reactions and the production of reactive oxygen species (ROS). Such a situation might be of importance in diseases such as thalassemia, where there is an abundance of iron-loaded transferrin in circulation.[17]

This appears to be the first report of interactions between transferrin fibres and human erythrocytes. The timescale observed here in vitro is quite short: about 4–5 h. In vivo, RBCs have a long lifetime of about 120 days. As yet there appear to be no reports of transferrin fibres being formed in the body, although the higher concentrations in the blood relative to those used here might favour fibril formation. Fibril formation in the body might require surfaces with particular compositions, such as mineral deposits or modified membranes, and would be worthy of further investigation.

There might be mechanisms in the body that prevent fibrilisation of transferrin in the blood, thereby mitigating its harmful interaction with erythrocytes. Investigating this mechanism might also yield important insight into preventing fibrilisation of proteins that are already known to be linked to diseases, thus resulting in new approaches to combat diseases aggravated by fibrillar protein aggregates such as Alzheimer's disease, Parkinson's disease and type II diabetes.[18]

## Conclusion

Deposition of solutions of human serum holo(Fe_2_)-transferrin on formvar surfaces can lead to the formation of long pea-pod-like structures attributable to bacterial contamination and the presence of PHB storage granules, under the nutrient-limiting conditions used. Such deposits were not observed for apo- or partially saturated transferrin, in which iron availability is more limited.

Interestingly, the pea-pod-like structures contained periodic nanomineralisation sites for uranyl stain, and extended incubation gave rise to extensive transferrin fibril structures. Optical microscopy and AFM studies showed that red blood cells readily adhere to these fibrils, which appeared to penetrate RBC membranes and to induce rapid cell destruction. In future work it will be interesting to investigate the conditions under which transferrin fibrils might form in vivo, because such fibrils might have physiological effects relevant to diseases.

## Experimental Section

**Materials**: All the solutions used were sterile. The solutions used were all passed through Chelex resin prior to sterilisation. Chelex resin, 12 000 MWCO dialysis membrane, NaHCO_3_, (NH_4_)_2_CO_3_ and human serum transferrins [T4132 (**1**), T1147 (**2**), T3400 (**3**), T0665 (**4**), T3400 (**5**), T6549 (**6**)] were purchased from Sigma. NuPAGE Novex 12 % Bis-Tris Gels (1.0 mm thick, 10-well) and NuPAGE MOPS SDS running buffer (20×) were purchased from Invitrogen. The carbon-formvar coated 200 mesh copper grids were purchased from Agar scientific, UK.

**General sample preparation method**: Stock solutions of hTf (10 μm) were prepared in a sterile NaHCO_3_ solution (10 mm), and then this stock was dialysed once against a sterile solution of NaHCO_3_ (10 mm) by using a 12 000 MWCO membrane. Due care was taken to dialyse the apo- and holo-Tf separately so that no metal exchange could take place. The dialysis was carried out at room temperature (24—27 °C). Protein concentrations in the dialysed solutions were determined from the known molar extinction coefficients for transferrin: *ε*_280_=103 900 cm^−1^ m^−1^ for diferric-hTf and 84 000 cm^−1^ m^−1^ for apo-hTf.[19] The stock solutions were diluted to the necessary concentration for the TEM experiments. The solutions used to dissolve the protein were sterile, but after dissolving of the lyophilised protein the resulting solutions were not passed through any sterile filter membrane or sterilised by any other method.

**Sample preparation for electron microscopy**: The stock solution of hTf was diluted to 3 μm in bicarbonate solution (pH 7.4, 3 mm) and incubated at 37 °C from 0–3 days. The TEM images were recorded by taking aliquots (8 μL) after 24, 48 and 72 h intervals during this incubation period [sample codes: T4132 (**1**), T1147 (**2**), T3400 (**3**), T0665 (**4**), T3400 (**5**), T6549 (**6**)]. The loading of the sample onto carbon-coated formvar surfaces, staining and drying were done inside a class-I biosafety cabinet. Aliquots (8 μL) were loaded onto carbon-formvar-coated copper grids and dried. After drying, the samples were negatively stained with uranyl acetate solution (2 %) for 30 s, and the excess uranyl acetate solution was absorbed by capillary action on a filter paper. Water (8 μL) was added, and again after 3 min the drop was blotted off as earlier. The water washing steps were repeated twice to wash out excess salt and stain from the samples, and the air-dried samples were stored in a desiccator for use in TEM experiments. Sample **2** was used for TEM after reduction with DTT (dithiothreitol; see sample preparation section for electrophoresis). The resulting sample **2** was diluted to 3 μm in NaHCO_3_ (3 mm), and 7 μL was spotted onto a carbon-coated formvar surface (300 mesh Cu grid) and washed and then stained as described in the sample preparation section for electron microscopy.

The one-week-old buffer sample on which the TEM was performed was prepared by using a 6 μL drop of a 1000-times-diluted 10 mm physiologically relevant buffer of pH 7.4 [buffer ingredients: disodium hydrogen phosphate (10 mm), NaHCO_3_ (5 mm), KCl (10 mm), MgCl_2_ (1 mm), pH adjusted to 7.4 with HCl (1 m)] and incubated at 25 °C for a week.

**Electrophoresis**: SDS-PAGE studies were performed with denatured protein samples without the use of any reducing agent (i.e., DTT) during denaturation. Denaturation was carried out by heating the protein solution at 60 °C for 5 min. Samples were loaded on Novex 12 % Bis**⋅**Tris gels. Electrophoresis was carried out with 1× MOPS-SDS buffer at 125 mV for 45 min. The reduction of sample **2** was carried out with DTT (60 mol equiv.) in apo-hTF solution (**2**, T1147 Sigma, 10 μm, 1 mL) made up in the physiologically relevant buffer (pH 7.4, 10 mm). The mixture was then incubated at 37 °C for a day, followed by centrifugation with a 10 000 MWCO centrifugal filter at 4 °C, and washed with NaHCO_3_ solution (5×3 mm, Figure S1).

**Incubation on LB agar plates**: Sterile LB agar plates were made by a standard procedure with sterile LB agar broth without any added antibiotics. These plates were used for testing the protein samples for bacterial contamination. Samples **1**–**6** [hTf solution (3 μm) in NaHCO_3_ (3 mm), 50 μL] were streaked on the LB agar plates and incubated at 37 °C for up to 48 h.

**Isolation of red blood cells**: Blood samples were collected from healthy human volunteers with informed consent. The blood samples were centrifuged at 2800 *g* for about 10–15 min at room temperature. Plasma and buffy coat were removed after each centrifugation. This was repeated about 3–4 times, and then the blood cells were suspended in phosphate-buffered saline solution [PBS, pH 7.4, NaCl (137 mm), KCl (2.7 mm), Na_2_HPO_4_ (4.3 mm), NaH_2_PO_4_ (1.47 mm)]. The isolated red blood cells were stored at 4 °C suspended in PBS solution.

**Immobilisation of red blood cells on surfaces**: Several methods to immobilise red blood cells on glass or mica surfaces have been developed. Most of them involve chemical fixing with glutaraldehyde solution (1 %). To obtain an image free from artefacts, we followed the method of an earlier report with small modifications.[18] An aliquot of the blood cells in PBS solution (5 μL) was manually spread on a clean microscope glass coverslip to form a thin blood film. The samples were air-fixed by vigorous manual gesticulation and then dried under dust-free conditions at room temperature. The regions of interest without any overlap with neighbouring cells were marked and visualised under an optical microscope.

**Transferrin binding to erythrocytes**: Transferrin solutions (12.5 μm) in sodium bicarbonate buffer were incubated with blood cells at 5 % hematocrit and an optimum temperature of 37 °C.

**Optical microscopy**: An aliquot of blood cells (2–5 μL) in PBS solution was spread onto the surfaces of clean microscope glass coverslips. The samples were dried at room temperature and viewed under an optical microscope (Leica DM2500M). Images obtained were electronically captured and transferred to computer with the aid of Leica Application Suite software.

**Atomic force microscopy**: Microscope glass coverslips were washed with detergent and water and then dried. An aliquot of the blood cells in PBS solution was spread on to the glass surface to form a thin blood film. The samples were air-fixed by vigorous manual gesticulation and then dried at room temperature. The regions of interest were marked under an optical microscope. Atomic force microscopy was carried out in air with an Agilent Technologies AFM (5500 AFM/SPM) operating in contact mode. The glass slide bearing the blood film was mounted on the XY stage of the AFM, and the integral video camera (NAVITAR, Model N9451A-USO6310233 with the Fiber-light source, MI-150 high-intensity illuminator from Dolan–Jenner Industries) was used. Silicon nitride cantilevers with resonant frequency of 87 kHz were used. The average dimension thickness, width and length of cantilever were about 2.0, 51 and 446 μm, respectively. The scanner model N9524A-USO7480132.xml/N9520A-USO7480152.xml was calibrated and used for imaging. The images were taken at room temperature in air with a scan speed of 2.0 lines s^−1^. Data acquisition and analysis were carried out with PicoView 1.8.2 and Pico Image Basic software, respectively.

**Transmission electron microscopy**: The transmission electron microscopy studies were performed with a JEOL 1200FX-II transmission electron microscope with an operating voltage of 80–100 kV. Carbon-formvar-coated 200 mesh copper grids, purchased from Agar scientific, UK, were used as received. The samples were drop cast onto the grids and dried in a bio safety cabinet (level-I).

Samples were imaged under bright-field conditions with an inserted objective aperture large enough to encompass the 3.44 Å main diffraction ring from the carbon-formvar grid. The dominant conditions for the image contrast obtained from the transferrins were of a kinematic nature and resulted either from variance in the sample thickness along the typical beam path or from expected variances in the atomic number (Z-contrast) within the transferrin structure.

EDX analysis was performed in standard TEM mode, but with employment of a convergent probe and a selection of small probe sizes more suitable to microanalysis, particularly on the darker structures noted within the transferrins.

## References

[1] Steere AN, Byrne SL, Chasteen ND, Mason AB (2012). Biochim. Biophys. Acta Gen. Subjects.

[2a] Booyjzsen C, Scarff CA, Moreton B, Portman I, Scrivens JH, Costantini G, Sadler PJ Biochim. Biophys. Acta Gen. Subjects.

[2b] Mahmoudi M, Quinlan-Pluck F, Monopoli MP, Sheibani S, Vali H, Dawson KA, Lynch I (2012). ACS Chem. Neurosci.

[2c] Jeppesen MD, Westh P, Otzen DE (2013). FEBS Lett.

[2d] Macchi F, Eisenkolb M, Kiefer H, Otzen DE (2010). Int. J. Mol. Sci.

[2e] Pal P, Mahato M, Kamilya T, Tah B, Sarkar R, Talapatra GB (2012). J. Phys. Chem. B.

[2f] Colvin VL, Kulinowski KM (2011). Proc. Natl. Acad. Sci. USA.

[3] Ghosh S, Mukherjee A, Sadler PJ, Verma S Angew. Chem. Int. Ed.

[4a] Flärdh K, Buttner MJ Nat. Rev. Microbiol.

[4b] Sudesh K, Abe H, Doi Y (2009). Prog. Polym. Sci.

[4c] Zhao XQ, Li WJ, Jiao WC, Li Y, Yuan WJ, Zhang YQ, Klenk HP, Suh JW, Bai FW (2000). Int. J. Syst. Evol. Microbiol.

[4d] Veening JW, Stewart EJ, Berngruber TW, Taddei F, Kuipers OP, Hamoen LW (2009). Proc. Natl. Acad. Sci. USA.

[4e] Du L, Jiang H, Liu X, Wang E (2008). Electrochem. Commun.

[5a] Morgan EH Mol. Aspects Med.

[5b] Iacopetta BJ, Morgan EH (1981). J. Biol. Chem.

[6] Van Renswoude J, Bridges KR, Harford JB, Klausner RD (1982). Proc. Natl. Acad. Sci. USA.

[7] Dautry-Varsat A, Ciechanover A, Lodish HF (1983). Proc. Natl. Acad. Sci. USA.

[8] Klinken SP (2002). Int. J. Biochem. Cell Biol.

[9a] Rasia M, Bollini A Biochim. Biophys. Acta Biomembr.

[9b] Joshi KB, Venkatesh V, Verma S (1998). Chem. Commun.

[10a] Lokesh BE, Abdul Hamid ZA, Arai T, Kosugi A, Murata Y, Hashim R, Sulaiman O, Mori Y, Sudesh K Clean Soil Air Water.

[10b] Spring S, Wagner M, Schumann P, Kaempfer P (2012). Int. J. Syst. Evol. Microbiol.

[11] Hedlund BP, Geiselbrecht AD, Bair TJ, Staley JT (1999). Appl. Environ. Microbiol.

[12] Kovacs AT, van Hartskamp M, Kuipers OP, van Kranenburg R (2010). Appl. Environ. Microbiol.

[13] Shleeva M, Mukamolova GV, Young M, Williams HD, Kaprelyants AS (2004). Microbiology.

[14] Varela C, Mauriaca C, Paradela A, Albar JP, Jerez CA, Chavez FP (2010). BMC Microbiol.

[15a] Haley KP, Skaar EP Microbes Infect.

[15b] Schryvers AB, Gonzalez GC (2012). Can. J. Microbiol.

[15c] Moraes TF, Yu RH, Strynadka NCJ, Schryvers AB (1990). Mol. Cell.

[15d] Williams P, Griffiths E (2009). Med. Microbiol. Immunol.

[16a] Tokiwa Y, Pranamuda H Biopolymers.

[16b] Saito T, Kobayashi T (2001). Biopolymers.

[16c] Lee SY, Park SH, Hong SH, Lee Y, Lee SH (2001). Biopolymers.

[17] Kadowaki H, Nishitoh H, Urano F, Sadamitsu C, Matsuzawa A, Takeda K, Masutani H, Yodoi J, Urano Y, Nagano T, Ichijo H (2005). Cell Death Differ.

[18] Irvine GB, El-Agnaf OM, Shankar GM, Walsh DM (2008). Mol. Med.

[19] James NG, Mason AB (2008). Anal. Biochem.

[20a] O'Reilly M, McDonnell L, O'Mullane J J. Ultramicroscopy.

[20b] Chen Y, Cai J (2001). Micron.

