# Supplementary material for: Human Serum Transferrin Fibrils: Nanomineralisation in Bacteria and Destruction of Red Blood Cells
Source: Chembiochem. 2014 Dec 4;16(1):149–55. doi: 10.1002/cbic.201402458 (PMC4371634; doi:10.1002/cbic.201402458)
Supplement: Supplementary file 1 [file cbic0016-0149-sd1.pdf]

CHEMBIOCHEM

Supporting Information

© Copyright Wiley-VCH Verlag GmbH & Co. KGaA, 69451 Weinheim, 2014

Human Serum Transferrin Fibrils: Nanomineralisation in Bacteria and Destruction of Red Blood Cells

Arindam Mukherjee,[a] Mark A. Barnett,[a] V. Venkatesh,[b] Sandeep Verma,*[b] and Peter J. Sadler*[a]

cbic_201402458_sm_miscellaneous_information.pdf

Supporting information

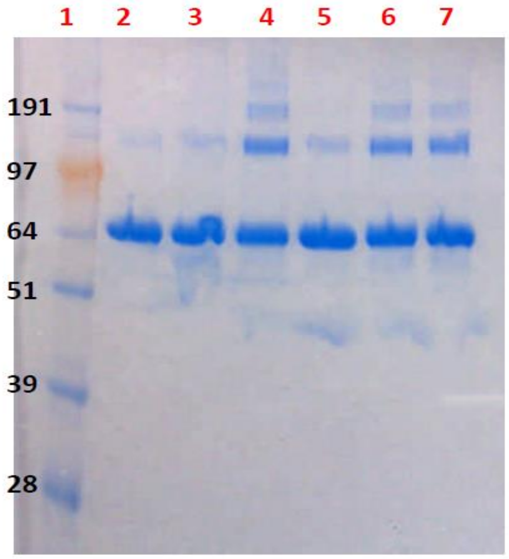

Figure S1. SDS PAGE of extensively dialyzed hTf samples **1**-**6** in 5 mM $NaHCO_3$ using 12% NuPAGE precast gel. No reducing agent was used. Lane 1 is the molecular weight marker and the respective molecular weights are labelled in the picture. Lane 2- holo transferrin (T4132/038K1107), Lane 3- apo-transferrin (T1147/068K1571), Lane 4- holo transferrin (T3400/68F_9468), Lane 5- holo transferrin (lot no. 0665/107K1182), Lane 6- human transferrin (T6549/24H9310), Lane 7- human transferrin (T3400/14H9320).

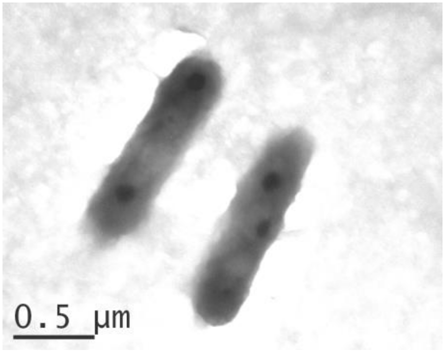

Figure S2. Sample **1** (T4132) showing bacteria like shapes on 0-48 h incubation at 37 °C in 3 mM $NaHCO_3$.

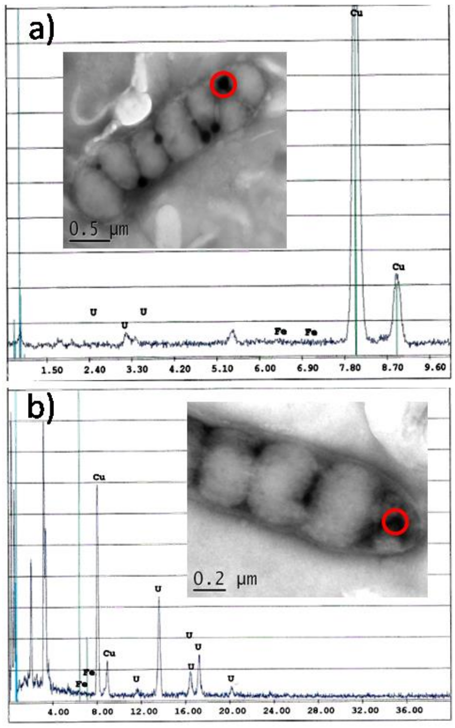

Figure S3. Energy dispersive X-ray plots of the periodic dark bands and spots (marked as red circles) from holotransferrin sample **3** (T3400, Sigma) showing that iron was not detected in either the bands or the dark spots, (green lines in b).

[a] Dr.Arindam Mukherjee, Dr Mark Barnett and Prof.Dr Peter J. Sadler
Department of Chemistry
University of Warwick
Gibbet Hill Road, Coventry CV4 7AL, UK
Fax: (+44) 024 76523819
E-mail: P.J.Sadler@warwick.ac.uk

[b] V. Venkateshand Prof. Dr Sandeep Verma
Department of Chemistry, Indian Institute of Technology-Kanpur
Kanpur 208016 (UP) (India)
Fax: (+91) 512-259-7436
E-mail: sverma@iitk.ac.in

Supporting information for this article is available on the WWW under http://www.chembiochem.org or from the author.

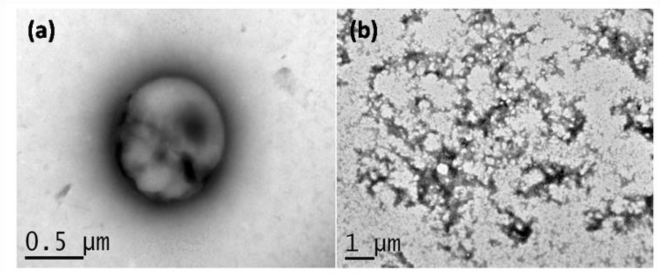

Figure S4. TEM images of sample **3**,holotransferrin (3 µM in 3 mM $NaHCO_3$) after incubation for 24-72 h at 60 °C, showing no tubular shapes ,rather globule like almost spherical shapes along with fine networked fibrillar-type precipitates.

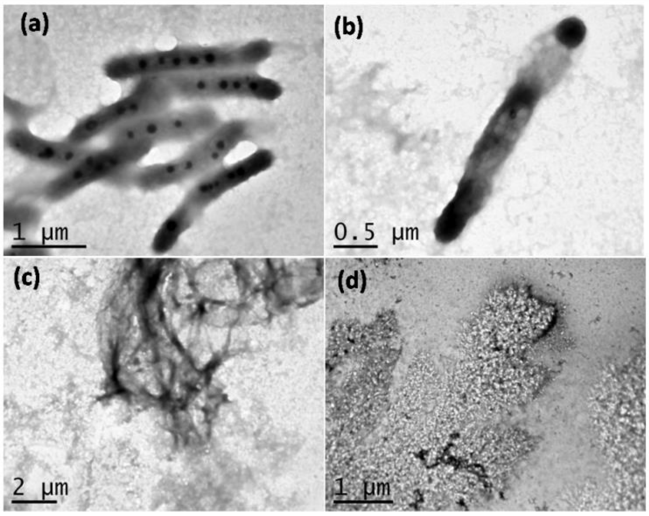

Figure S5. 3 uM samples of **4** in 3 mM $NaHCO_3$, pH 7.4, incubated at 37 °C for 24-48 h. Top- bacteria like shapes with periodic dots, but unlike sample **3** and **6** ;Bottom- some protein precipitates with very fine fibrous-type branching and thin fibrous overlapping shapes, but the thin fibres (bottom right) are very low in contrast.

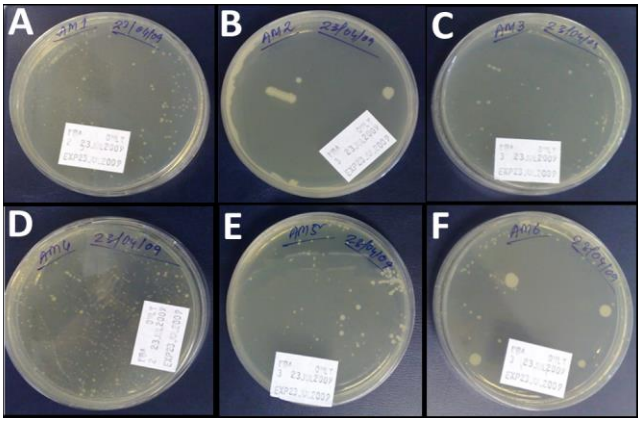

Figure S6. Bacterial growth in samples of transferrin used in TEM, plated onto LB agar plates and incubated at 37°C with no antibiotics. Bacterial growth was observed for sample **1**,**3** and after 18 h (A , C and F), in **2** and **5** after 36 h (B and E), for **4** after 48 h (D), in the same incubator.

6

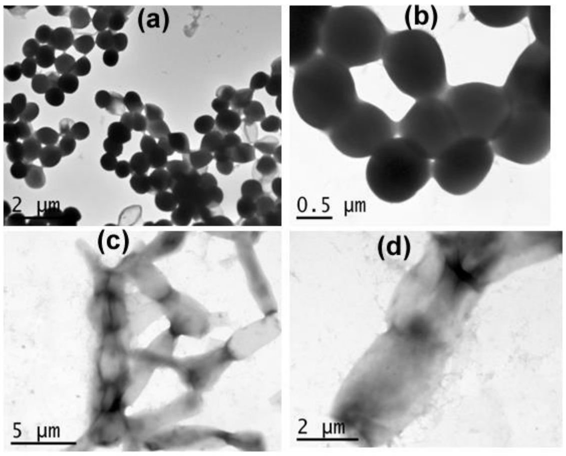

Figure S7. Representative TEM images of bacterial colonies picked from the LB agar plates of transferrin samples **1**-**6** incubated at 37°C (a and b) and a non-sterile physiological buffer solution incubated for 7 days at 37 °C (c and d).

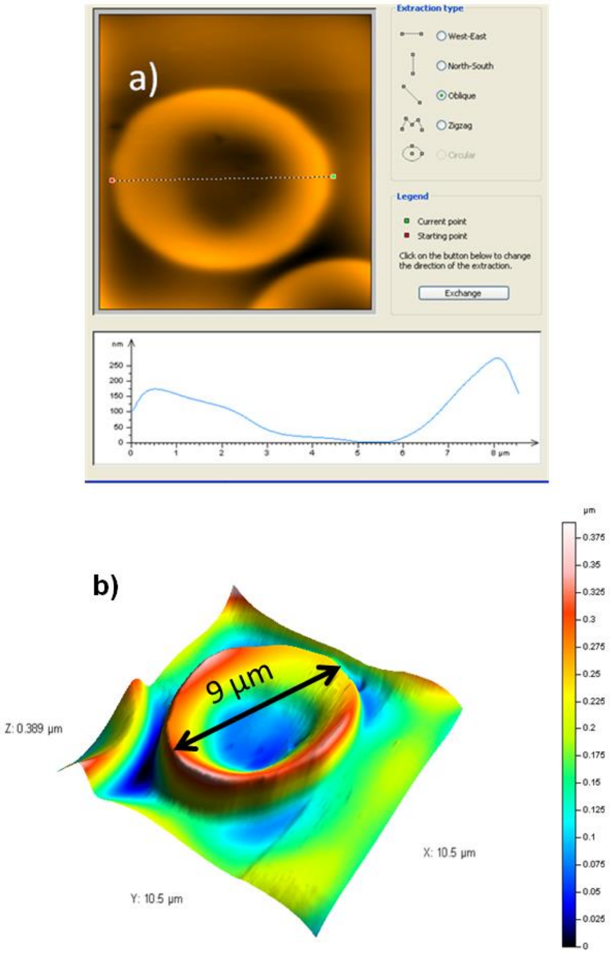

Figure S8. a) AFM height profile; b) 3-D Colour contour of a fresh human erythrocyte.

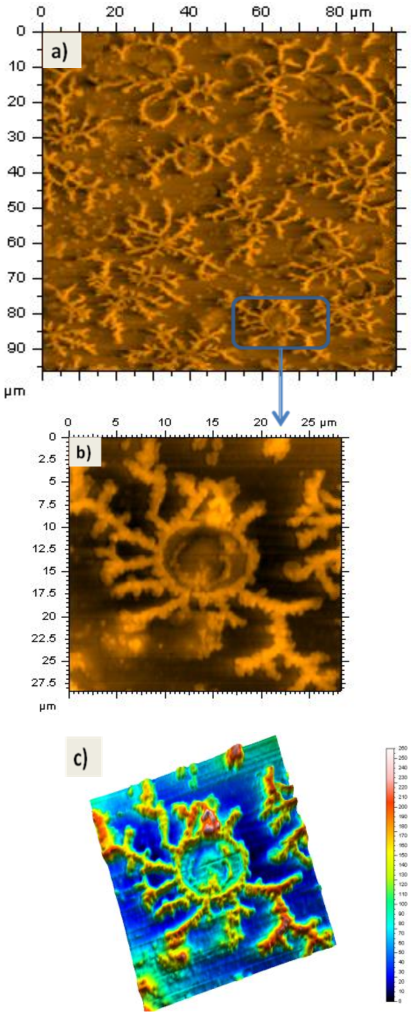

Figure S9: Contact mode AFM micrograph images of (a) Sample **3** holo-transferrin solutions incubated with erythrocytes under fresh conditions; (b) Magnified region from (a); (c) 3D micrograph of erythrocyte-bound transferrin with *z* scale.
